# Multifocal Primary Neoplasms in Kidney Allografts: Evaluation of Two Cases

**DOI:** 10.15586/jkcvhl.2016.53

**Published:** 2016-06-13

**Authors:** Robert J. Ellis, Keng Lim Ng, Hemamali Samaratunga, Sharon J. Del Vecchio, Simon T. Wood, Glenda C Gobe

**Affiliations:** 1 Centre for Kidney Disease Research, School of Medicine, University of Queensland, Brisbane, Australia; 2 Department of Urology, Princess Alexandra Hospital, Brisbane, Australia; 3 Department of Surgery, University Malaya, Kuala Lumpur, Malaysia; 4 Aquesta Pathology, Brisbane, Australia

**Keywords:** multifocal, renal allograft, renal cell carcinoma, renal neoplasm

## Abstract

Renal cell carcinoma (RCC) is the fifth most common malignancy in kidney transplant recipients, with increased risk arising due to immunosuppression. *De novo* RCC occurrence in kidney allografts is much less common when compared with the native kidneys. Multifocal RCC in allograft kidneys is rarely described. In this report, we discuss two cases of *de novo* multifocal renal neoplasms in allograft kidneys. Case 1 had three distinct neoplastic lesions of >5 mm, and case 2 had four. Using the World Health Organization 2016 classification of adult renal tumours, case 1 had one clear-cell (cc) RCC (grade 3) and two papillary adenomas; all confined to the kidney. Case 2 had a nodular lesion classified as ccRCC (grade 4) with focal rhabdoid differentiation and some infiltration of renal sinus fat; a cc tubulopapillary RCC; a multilocular cystic renal neoplasm of low malignant potential; and a mucinous tubular and spindle cell carcinoma; the last three all confined to the kidney. This is the first report of mucinous tubular and spindle cell carcinoma in a kidney allograft. When considering multifocal RCC with discordant histology, it is likely that these represent independent tumourigenic events.

## Introduction

Transplant recipients are at a substantially increased risk of developing malignancy due to immunosuppression regimens necessary to maintain graft viability. This may be due to decreased tumour antigen surveillance and/or increased risk of oncogenic infections (e.g. Epstein-Barr virus) (1). Renal cell carcinoma (RCC) is the fifth most common malignancy in kidney transplant recipients, and there is an estimated 15-fold increased risk when compared with the general population (2). The majority of these cancers arise in native kidneys, where end-stage or acquired cystic parenchymal changes often compound cancer risk, in conjunction with immunosuppression. Malignancies arising in allografts are substantially less common.

Reports of multifocal RCC in allograft kidneys are sparse although multifocal RCC has been reported in 5–25% of all sporadic RCC cases (3). Synchronous multifocal RCC is a common finding in association with many hereditary conditions (e.g. von Hippel-Lindau [VHL] disease and Birt-Hogg-Dubé syndrome); however, outside these conditions, there is a conflicting evidence as to exact pathogenic mechanisms, namely, whether tumours arise independently or as a result of intrarenal metastasis. When considering multifocal RCC with discordant histology, it is likely that these represent independent tumorigenic events (4). Although concomitant tumours have been reported following kidney transplantation, they are generally spatiotemporally demarcated (5). This report details two cases of multifocal *de novo* RCC arising in kidney allografts, with particular focus on the pathological findings. In both cases, there were multiple co-existing renal neoplasms with variable histological features within the graft kidney.

## Patients and methods

### Ethics approval

Relevant approvals were granted by institutional ethics review boards. Written informed consent was obtained prior to patient inclusion.

### Patients

Retrospective analysis was undertaken reviewing the records of 183 patients who underwent tumour nephrectomy at the Princess Alexandra Hospital (Queensland, Australia) between June 2013 and December 2015. We identified nine of these patients as being kidney transplant recipients. Of these nine, two had lesions within their allograft kidney. Both patients received conventional transplant nephrectomy.

### Case 1

**Figure 1.**
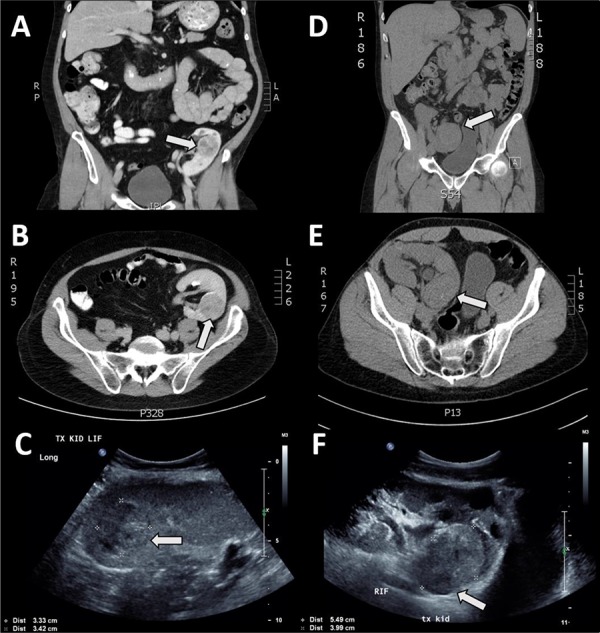
**A**. Computer tomographic scan (CT) case 1: coronal section showing transplant kidney in left iliac fossa; distinct mass present in upper pole and enhanced with contrast, designated by arrow. **B**. CT case 1: transverse section showing distinct mass in transplant kidney enhanced with contrast, predominantly posteriorly located. **C**. Ultrasound case 1: showing distinct mass within kidney. **D**. CT case 2, coronal section showing transplant kidney in right iliac fossa; indiscriminate mass located in lower pole without contrast enhancement. **E**. CT case 2, transverse section showing indiscriminate mass in posterior portion of kidney. **F**. Ultrasound case 2, showing distinct mass in posterior portion of kidney cortex.

**Figure 2.**
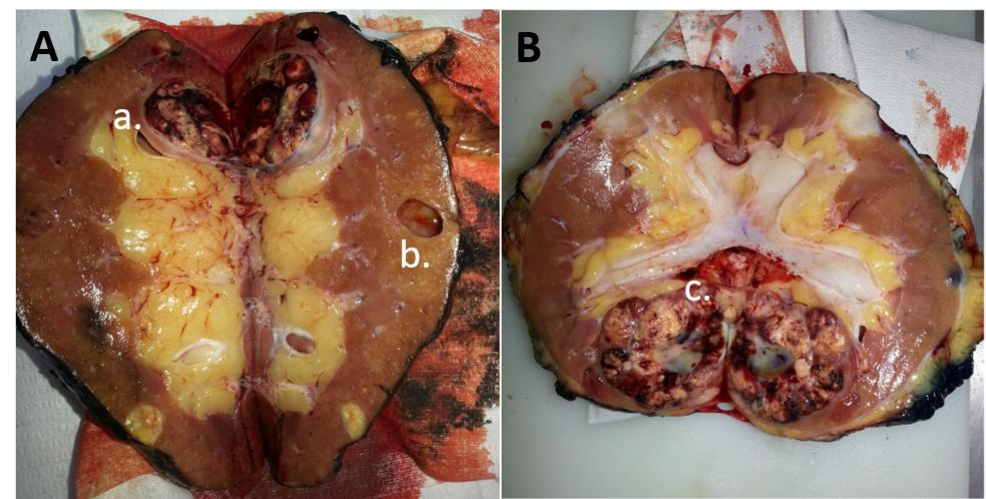
**A**. Bivalved allograft kidney, case 1: Primary lesion is clear-cell renal cell carcinoma (ccRCC) visible in upper pole (a.). Papillary adenomas not easily visualised. Cortical cyst visible (b.). **B.** Bivalved allograft kidney, case 2 : primary lesion (ccRCC) visible in lower pole (c.); note invasion into renal sinus fat.

A 55-year-old man underwent graft nephrectomy after a solid mass lesion was detected in his transplanted kidney on ultrasound and confirmed by computed tomographic (CT) scan (**Figure 1A-C**). The kidney transplant was performed in 1997 following end-stage kidney disease due to focal segmental glomerulosclerosis (18-year functioning graft; estimated glomerular filtration rate [eGFR] prior to surgery was 45 ml/min per 1.73 m^2^). Immunosuppressive therapeutic regimen prior to surgery included prednisolone (5 mg/day), mycophenolate mofetil (2000 mg/day) and tacrolimus (0.5 mg/day). In the non-neoplastic kidney sections, there were glomerular hypertrophy and focal segmental glomerulosclerosis, with no transplant glomerulopathy. Mild transplant arteriopathy was present, and there was minimal tubular atrophy/interstitial fibrosis (<10% cortex). There was no evidence of rejection. Multiple simple cortical cysts (**Figure 2A**) and papillary adenomas (<5 mm) were present.

**Figure 3.**
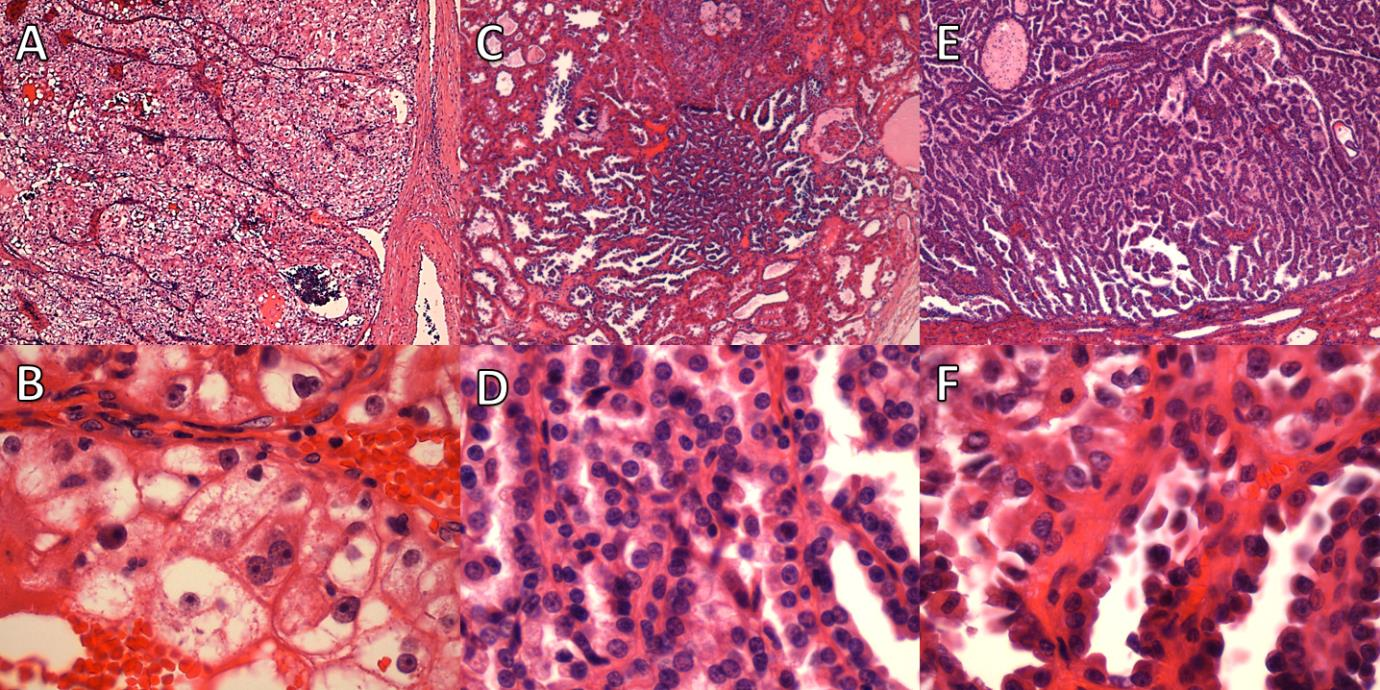
Case 1: **A** and **B**. Clear-cell renal cell carcinoma, grade 3; high- and low-power fields of the two papillary adenomas are shown in **C** and **D** and **E** and **F.**

The following lesions were also found in the kidney: a nodular lesion present in the upper pole (35 mm at largest dimension) classified as clear-cell (cc) RCC (WHO/ISUP grade 3); and two additional nodules (8 and 9 mm at largest dimension) were identified in the lower pole, both classified as papillary adenomas. Both of these lesions were initially classified as type 1 papillary RCC (grade 2); however, these lesions were reclassified by a uropathologist as papillary adenomas, based on the World Health Organization 2016 classification of adult renal tumours (lesions are <15 mm at largest diameter) (6). All tumours were confined to the kidney (**Figure 3**).

### Case 2

A 44-year-old man underwent graft nephrectomy after a solid mass lesion was detected in his transplanted kidney on ultrasound, and confirmed by a CT scan (**Figure 1D-F**). The transplant was received in 1996 (19-year functioning graft; eGFR prior to surgery was 26 ml/min per 1.73 m^2^). Histology of the non-neoplastic kidney showed mild parenchymal scarring with some glomerulitis and transplant glomerulopathy present. Moderate transplant arteriopathy and hyaline arteriosclerosis were also present. Indications of active chronic antibody-mediated rejection were present in the microvessels (glomerular and peritubular capillaries) and arteries, as evidenced by C4d immunoperoxidase stain positivity. Multiple cortical cysts were present.

**Figure 4.**
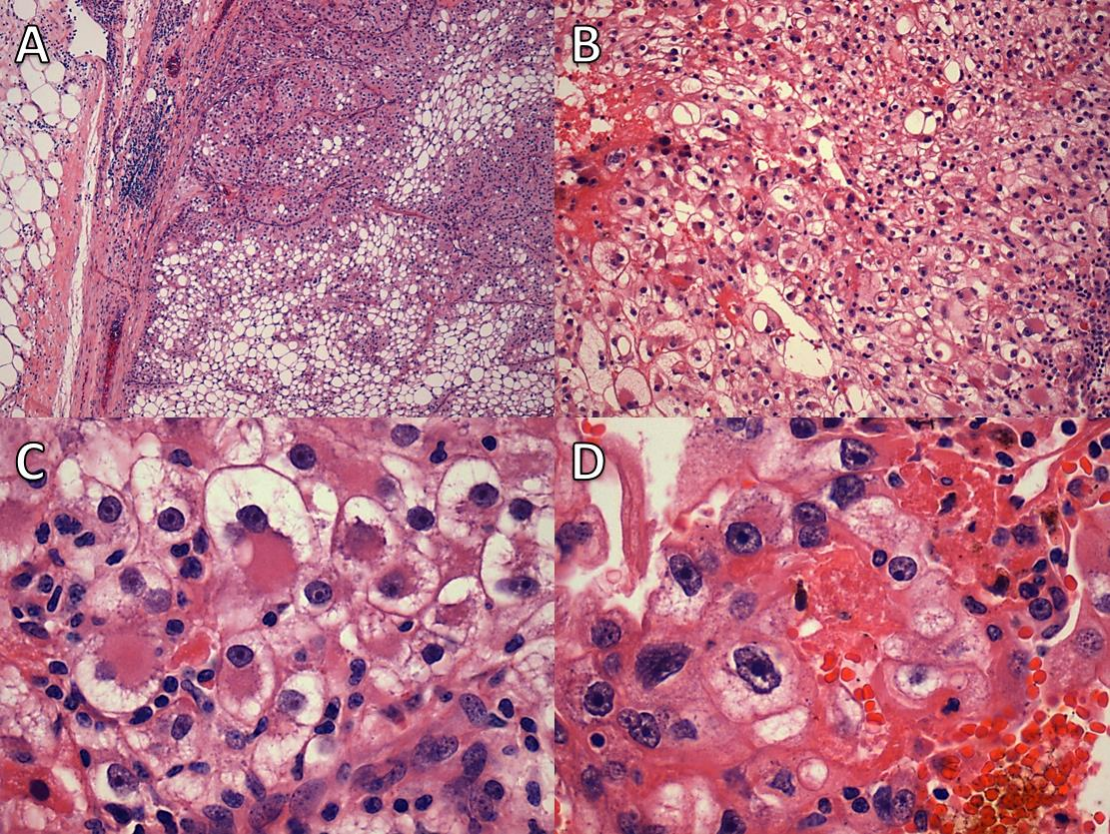
Case 2: **A** and **B**. Low-power fields showing clear cell renal cell carcinoma. **C**. High-power field showing rhabdoid differentiation. **D**. High-power field showing features consistent with a grade of 4.

**Figure 5.**
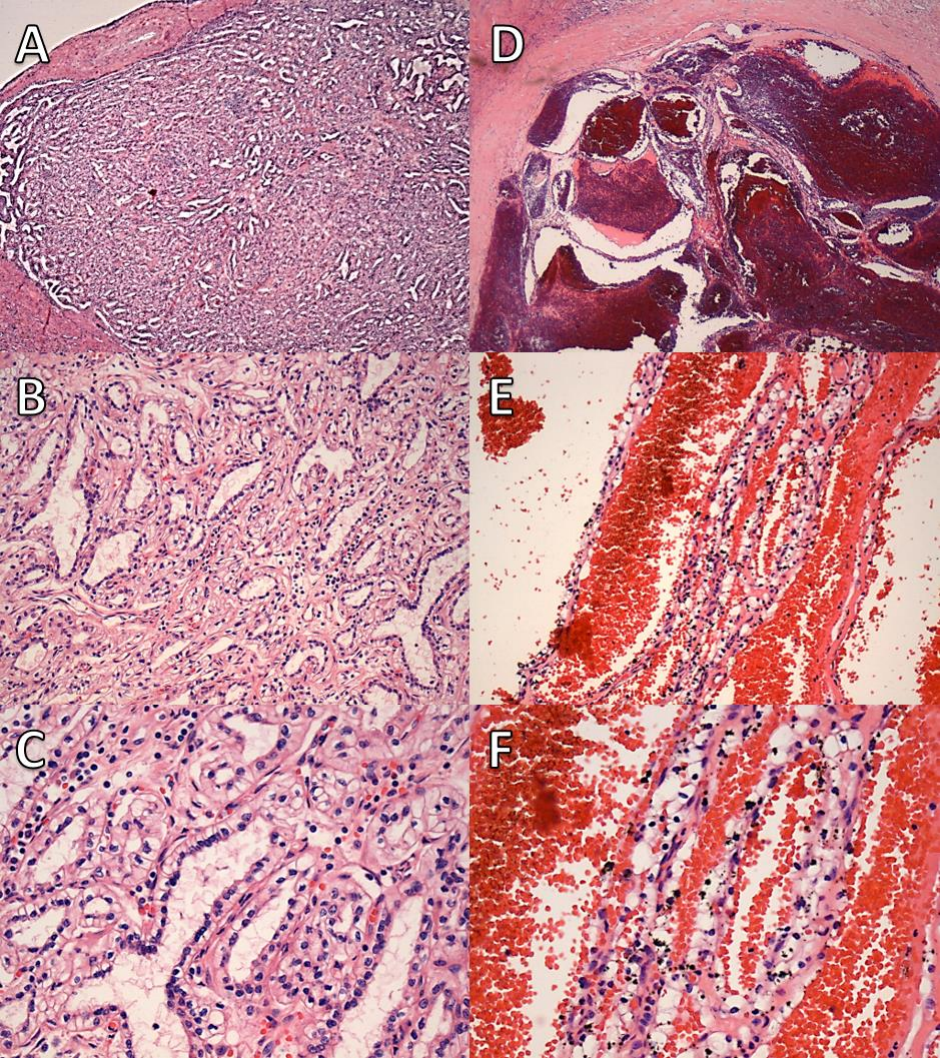
Case 2: **A-C**. Clear-cell tubulopapillary renal cell carcinoma. **D-F**. Multilocular cystic renal neoplasm of low malignant potential.

**Figure 6.**
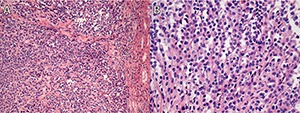
Case 2: **A**. Low-power and (**B**) high-power fields showing mucinous tubular and spindle cell carcinoma.

A nodular lesion present in the lower pole (50 mm at largest dimension) was classified as ccRCC (grade 4), with focal rhabdoid differentiation (**Figure 4**). This tumour showed some infiltration of renal sinus fat (**Figure 2B**). A second lesion in the lower pole (37 mm at largest diameter) was classified as a cc tubulopapillary RCC (low grade; **Figure 5A-C**). A third lesion in the lower pole (7 mm at largest dimension) was classified as multilocular cystic renal neoplasm of low malignant potential (**Figure 5D-F**). A 7-mm lesion in the upper pole was classified as mucinous tubular and spindle cell carcinoma (**Figure 6**). All tumours were limited to the kidney.

## Discussion

Here, we report two cases of multifocal synchronous neoplasms in allograft kidneys, with variable histological concordance. We found three similar case studies in the literature (7–9), and a case series identified a further 12 cases of multifocal RCC in allograft kidneys (10). Malignancies arising in allograft kidneys have three primary mechanisms of pathogenesis: (i) *de novo* malignancy, (ii) recurrence of previous malignancy (i.e. RCC) or (iii) transmission of malignancy from donor (11). In the absence of previous RCC history and considering the long life of both grafts, it can be presumed that both cases exhibit *de novo* malignancy (10). Although multifocal neoplasms in allograft kidneys are sparsely reported, a recent study of 2,569 nephrectomy patients revealed a 3% prevalence of ipsilateral multifocal synchronous RCC in the general population, with only 58.8% histological concordance (12). This indicates that with multifocal lesions, it is not uncommon for multiple histological subtypes to be found.

The predominant mass in both cases was ccRCC (summarised in **Table 1**). Although there is dispute as to whether multifocal ccRCC is the result of related or independent events (13, 14), there is no indication that they give rise to other subtypes. Considering case 1, it has been reported that multifocal papillary RCC generally arise independently (15). This tends to indicate that the multiple papillary adenomas probably arose from independent tumorigenic events, especially considering that multifocal papillary adenomas are not uncommon and often are associated with multifocal papillary RCC (15).

**Table 1.** Histological Diagnosis of Lesions

Histology	Size (mm)
Case 1	
Clear cell RCC	35
Papillary adenoma	8
Papillary adenoma	9
Case 2	
Clear-cell RCC	50
Clear-cell tubulopapillary RCC	37
Multilocular cystic renal neoplasm	7
Mucinous tubular and spindle cell carcinoma	7

Considering case 2, cc tubulopapillary RCC by definition do not exhibit 3p deletion, chromosome 7 or 17 polysomy, or *VHL* mutations, as is generally found in ccRCC (16). Based on this, polyclonal origin of these lesions is extremely likely. Of the three previous case studies of multifocal RCC in allografts, two showed discordant histological subtypes. The first case was of a high-grade ccRCC and papillary RCC (7), and the second showed 29 separate lesions with multiple classifications, predominantly papillary RCC or adenoma (9).

Evaluation of the cases presented in this report, and previously reported, provides some evidence that multiple tumorigenic mutations may lead to multifocal synchronous RCC occurrence in kidney allografts. Although immunosuppression presents as a common factor, it is not possible to determine whether it predisposes to multiple mutations, as causes are almost always multifactorial. Additional research to determine the relationship between immunosuppression and tumorigenic events is required.

There are important connotations regarding methods to diagnose allograft tumours, and further support for ongoing tumour screening in kidney transplant recipients with long graft life. First, as identified by Simhan et al. (12), if a single region of an intrarenal mass is biopsied and found to be of low malignant potential, there is no guarantee that there is not a concomitant high-grade tumour also present. The importance of thorough and appropriate radiological examination is also demonstrated because if multifocal lesions are present, there is a possibility that innocuous masses may be overlooked with subsequent progression (especially relevant in the context of nephron-sparing surgery). Difficulty in this regard can be compounded as radiographic contrast is often contraindicated in patients with diminishing graft kidney function (as the case in **Figure 1D-E**). Although there is not enough evidence to justify adjustments of diagnostic protocols at present, we do highlight the importance of thorough and accurate radiographic/histologic evaluation of patients with suspected kidney allograft neoplasia.

## Conclusion

Overall, we report two cases of multifocal allograft neoplasms with low histological concordance. Although our findings are limited by the lack of genetic analysis of tumour tissue, based on the available literature, it appears that they are the result of independent tumorigenic mutations as opposed to intrarenal metastatic spread. Although there is not enough evidence to implicate immunosuppression as the sole causative factor, further investigation into this link is warranted. Our findings highlight the importance of effective and thorough radiologic and histologic analyses of tumours present in kidney allografts.
